# In-vitro influence of mycophenolate mofetil (MMF) and Ciclosporin A (CsA) on cytokine induced killer (CIK) cell immunotherapy

**DOI:** 10.1186/s12967-016-1024-4

**Published:** 2016-09-13

**Authors:** Melanie Bremm, Sabine Huenecke, Olga Zimmermann, Verena Pfirrmann, Andrea Quaiser, Halvard Bonig, Jan Soerensen, Thomas Klingebiel, Eva Rettinger, Peter Bader, Claudia Cappel

**Affiliations:** 1 Clinic for Pediatric and Adolescent Medicine, University Hospital, Theodor-Stern-Kai 7, 60596 Frankfurt/Main, Germany; 2 Division for Translational Development of Cellular Therapeutics, Institute for Transfusion Medicine and Immunohematology, Goethe-University Frankfurt/Main, Frankfurt/Main, Germany; 3 German Red Cross Blood Donor Service Baden-Württemberg-Hessen, Frankfurt/Main, Germany

**Keywords:** MMF, MPA, CIK cells, Immunosuppressive therapy, Immunotherapy, Allogeneic stem cell transplantation

## Abstract

**Background:**

Cytokine-induced-killer (CIK) cells are a promising immunotherapeutic approach for impending relapse following hematopoietic stem cell transplantation (HSCT). However, there is a high risk for treatment failure associated with severe graft versus host disease (GvHD) necessitating pharmaceutical intervention post-transplant. Whether immunosuppression with mycophenolate mofetil (MMF) or Ciclosporin A (CsA) influences the cytotoxic effect of CIK cell immunotherapy is still an open issue.

**Methods:**

CIK cells were generated from PBMC as previously described followed by co-incubation with mycophenolic acid (MPA) or CsA. Proliferation, cytotoxicity and receptor expression were investigated following short- (24 h), intermediate- (3 days) and long-term (7 days) MPA incubation with the intention to simulate the in vivo situation when CIK cells were given to a patient with relevant MPA/CsA plasma levels.

**Results:**

Short-term MPA treatment led to unchanged proliferation capacity and barely had any effect on viability and cytotoxic capability in vitro. The composition of CIK cells with respect to T-, NK-like T- and NK cells remained stable. Intermediate MPA treatment lacked effects on NKG2D, FasL and TRAIL receptor expression, while an influence on proliferation and viability was detectable. Furthermore, long-term treatment significantly impaired proliferation, restricted viability and drastically reduced migration-relevant receptors accompanied by an alteration in the CD4/CD8 ratio. CD3^+^CD56^+^ cells upregulated receptors relevant for CIK cell killing and migration, whereas T cells showed the most interference through significant reductions in receptor expression. Interestingly, CsA treatment had no significant influence on CIK cell viability and the cytotoxic potential against K562.

**Conclusions:**

Our data indicate that if immunosuppressant therapy is indispensable, efficacy of CIK cells is maintained at least short-term, although more frequent dosing might be necessary.

**Electronic supplementary material:**

The online version of this article (doi:10.1186/s12967-016-1024-4) contains supplementary material, which is available to authorized users.

## Background

The transplantation of allogeneic hematopoietic stem cells (HSCT) is an established therapy option for the treatment of relapsed leukemia and other hematological disorders [1, 2].

For prevention and treatment of severe GvHD following HSCT, the immunosuppressive drug mycophenolate mofetil (MMF; Cellcept) and Ciclosporin A (CsA) may be administered [3]. MMF is a prodrug which is systemically metabolized to the active metabolite mycophenolic acid (MPA). MPA non-competitively inhibits inosine monophosphate dehydrogenase (IMPDH) which plays an important role in the de novo nucleotide synthesis. Thereby, MPA effectively inhibits the cell proliferation depending on de novo nucleotide synthesis [4–7]. CsA is a calcineurin inhibitor, which suppresses the activation of IL-2 transcription leading to a reduced immune response especially of T cells [8]. Patients with aGVHD >grade I and/or immunosuppression are not eligible for CIK cell therapy. Anyhow, relevant MPA plasma levels might still be present at the time of CIK cell treatment due to intra- and inter-patient variability. In addition, CIK cells may cause GvHD necessitating pharmaceutical intervention, which among others may include the administration of MMF. We previously investigated the influence of MMF on NK cells within the scope of a clinical phase I/II study where patients received IL-2 stimulated NK cell immunotherapy to target high-risk leukemia or tumors. In this evaluation we observed that short-term (24 h) MPA incubation had no or marginal effects on the phenotype and only moderately reduced cytotoxic capability of IL2-stimulated NK cells in contrast to unstimulated NK cells [9].

In an ongoing study we currently investigate the immunotherapy with cytokine induced killer (CIK) cells derived from peripheral blood mononuclear cells (PBMC) of the stem cell donor via stimulation with interferon (IFN)-γ, OKT-3, IL-2 and IL-15 over a period of 10–12 days [10–13]. CIK cells are a heterogeneous population primarily consisting of a minor contribution of CD3^−^CD56^+^ NK cells and a majority of CD3^+^CD56^−^ T cells and CD3^+^CD56^+^ NK-like T cells [14, 15]. The cytotoxic activity of CIK cells against several tumor cell lines including leukemia, lymphoma and solid tumors was shown [16–19]. Among CIK cells, CD3^+^CD56^+^ NK-like T cells, which are derived from CD3^+^CD56^−^ T cells acquiring the CD56 molecule during expansion, showed the strongest proliferation and cytotoxic potential [14, 20, 21]. In first clinical applications we and others showed the safety and feasibility of CIK cell immunotherapy, including their relatively low propensity for causing GvHD even in only partially MHC-matched recipients [14, 22, 23]. By now, IL-15 activated CIK cells have been licensed as an advanced medicinal product for patients with high-risk leukemia and myelodysplastic syndrome (ATMP § 4b Abs. 3 AMG, license number: PEI.A.11630.01.1) [24]. Whether immunosuppressive therapy influences the survival and cytotoxic effect of CIK cell immunotherapy remains an open issue. Therefore, we investigated the in vitro effect of short, intermediate and long-term MPA incubation in therapeutically relevant concentrations on CIK cells which were manufactured over a period of 10–12 days according to our study protocol.

## Methods

### CIK cell generation and cultivation

CIK cells were generated from underweight banked blood of healthy donors (IRB approval 329/10) by standard ficoll separation (Biochrom AG, Berlin Germany). PBMC were adjusted to 3 × 10^6^ cells/ml and cultured in X-VIVO 10 media (Lonza, Verviers, Belgium) supplemented with 10 % fresh frozen plasma (German Red Cross Blood Donor Service, Frankfurt, Germany) in cell culture flasks (Greiner, Nürtingen, Germany) at 37 °C and 5 % CO_2_. At day 0 of CIK generation 1000 U/ml IFN-γ (Imukin^®^, Boeringer Ingelheim Pharma, Germany) were added, followed by 100 ng/ml anti-CD3 mAB (OKT3, MACS GMP CD3 pure, Miltenyi Biotec, Bergisch Gladbach, Germany) and 500 U/ml IL-2 (Proleukin^®^S, Novartis Pharma, Nuremberg, Germany) 24 h later (day 1). On days 4 and 8 of culture, cell density was adjusted to 1 × 10^6^ cells/ml and cells were re-stimulated with 50 ng/ml IL-15 (PeproTech, Rocky Hill, USA) until they were harvested on day 10. CIK cells were split; control flasks were further supplemented only with 50 ng/ml IL-15 every 3 days, test flasks were additionally spiked with a therapeutically relevant MPA concentration of 10 µM (Sigma-Aldrich, Taufkirchen, Germany) or of 5 µg/ml Ciclosporin A (CsA, Sandimmun^®^, Novartis Pharma GmbH, Nuremberg) after harvesting. The long term series received a further MPA/CsA treatment 3 days following harvesting.

### Preparation for ex vivo investigations

**Fig. 1:**
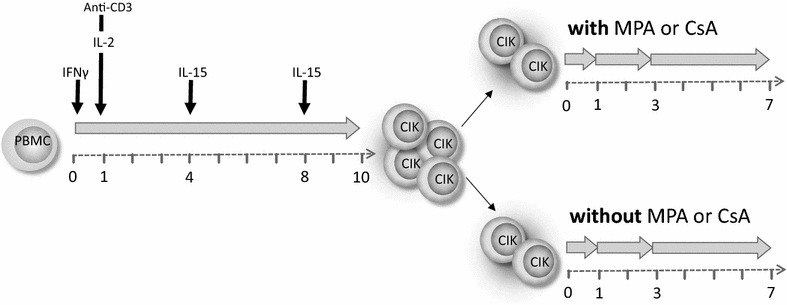
Experimental schedule of short-, intermediate- and long-term MPA/CsA incubation. CIK cells were generated by stimulation with interferon (IFN)-γ, OKT3, IL-2 and IL-15 over a period of 10 days. Subsequently, cells were split and either supplemented with 50 ng/ml IL-15 every 3 days (control) or additionally treated with 10 µM MPA or 5 µg/ml CsA. Proliferation, cytokine secretion and receptor expression were investigated following short- (24 h), intermediate- (3 days) and long-term (7 days) MPA treatment

For the experiments regarding cytotoxicity, surface receptor expression and cytokine/chemokine secretion, bench scale CIK cells of healthy donors were used (n = 6, independent experiments). Co-incubation experiments with/without MPA/CsA started after 10 days of cultivation. Proliferation, cytokine secretion and receptor expression was investigated following short- (24 h), intermediate- (3 days) and long-term (7 days) MPA treatment, whereas cytotoxicity was investigated following short- and intermediate-term MPA incubation, only (Fig. 1).

### Flow cytometric analyses

Flow cytometric analyses of CIK cells were performed on a Navios™ 10-color and a FC500 5-color flow cytometer (both Beckman Coulter, Krefeld, Germany). For cell staining monoclonal antibodies conjugated with fluorescein–isothiocyanate (FITC), phycoerythrin (PE), phycoerythrin-Texas Red^®^ (ECD), phycoerythrin–cyanine-5 and 5.5 (PC-5 and PC-5.5), phycoerythrin–cyanine-7 (PC-7), allophycocyanin (APC), APC-Alexa Fluor 700 (APC-A700), APC-Alexa Fluor 750 (APC-A750), Pacific Blue™ (PB), Krome Orange (KO) were used against following antigens (clones): FITC: TCRγδ (IMMU510), CD62L (DREG56), CD226/DNAM-1 (DX11), CD11a/LFA-1 (HI111)^2^, PE: TCRαβ (BW242/412)^1^, CD314/NKG2D (ON72)/(149810)^4^, CD173/CD95L/FAS-Ligand (NOK-1)^2^, CD183/CXCR3 (2D7)^2^, CD195/CCR5 (1C6)^2^, CD262/DR5/TRAIL (DJR2-4)^3^; ECD: CD19 (J3-119), CD45RO (UCHL1); PC-5.5: CD45 (J.33), CD45RO (UCHL1); PC-7: CD56 (N901/NKH-1); APC: CD3 (UCHT1), CD4 (13B8.2); APC-A700: CD25 (B1.49.9); APC-A750: CD16 (3GB), CD3 (UCHT1); PB: CD14 (RMO52), CD45RA (2H4); KO: CD45 (J.33), CD8 (B9.11) (all mouse IgG1, other than ^#^IgG2a, *IgG2b, all antibodies Beckman Coulter, except ^1^Miltenyi Biotec, ^2^BD Biosciences; ^3^Biolegend, ^4^R&D Systems). For the assessment of cell viability 7-AAD was used. Absolute counts were calculated via single-platform using Flow-Count™ fluorospheres (Beckman Coulter, Krefeld, Germany). All data were analyzed using CXP system II (Vs 2.2), Navios (Vs. 1.2) or Kaluza (Vs. 1.2) software (Beckman Coulter, Krefeld, Germany).

### Cytotoxicity assay

CIK cell cytotoxicity was tested against THP-1, K562 and MOLT-4 using the non-radioactive europium release cytotoxicity assay as described previously [16, 20]. To avoid the known discharge of the labeling reagent BATDA, K562 were previously incubated with Probenecid (Sigma-Aldrich Chemie GmbH, Steinheim, Germany). Target cells and CIK effector cells were co-cultured in triplicates at effector to target (E:T) ratios 40:1, 20:1, 10:1 and 5:1 on U-bottomed 96-well culture plates (Nunclon™, Thermo Fisher Scientific, Roskilde, Denmark). After 3 h co-incubation at 37 °C, 20 µl supernatant was collected from each well and incubated for 15 min (shaking, 250 rpm) with 200 µl Europium solution (PerkinElmer, Boston, USA). The fluorescence signal, correlating with the amount of destroyed cells, was then measured by a multilabel plate reader (VICTOR^3^™ 1420 multilabel counter, PerkinElmer, Boston USA). Target cells without effector cells were used as negative control. Maximum release (positive control) was obtained by target cell incubation with 16 % Triton™ X-100 solution (Sigma-Aldrich Chemie, Steinheim, Germany). Percentage of specific cytotoxicity was defined as the loss of target cells in relation to the mono-cultured control.

### Cytokine/chemokine analysis

Supernatants of expanded CIK cells after 10 days of cultivation and then following 24 h, 3 and 7 days MPA incubation (10 µM) with respective controls were collected and assayed using BioLegend LEGENDplex™ (BioLegend, San Diego, USA). Data acquisition was performed on a Navios Flow Cytometer and analyzed with the LEGENDplex™ Data Analysis Software (BioLegend, San Diego, USA). The cell density was adjusted to 3 × 10^6^/ml. The human inflammation 13-plex panel was designed for quantification of the cytokines/chemokines IL-1β, IFN-α, IFN-γ, TNF-α, MCP-1/CCL2, IL-6, IL-8, IL-10, IL-12p70, IL-17A, IL-18, IL-23 and IL-33. The minimum detectable concentration for the cytokines ranged from 0.6 to 2.1 pg/ml.

### Statistical analysis

Statistical analysis was performed using GraphPad Prism 6 for Windows (GraphPad Software, San Diego, USA). Data were compared by paired, non-parametric Friedmann test and differences were considered as significant for p < 0.05 (*), p < 0.01 (**) and p < 0.001 (***).

## Results

### Influence of MPA/CsA treatment on CIK cell expansion and viability

**Fig. 2:**
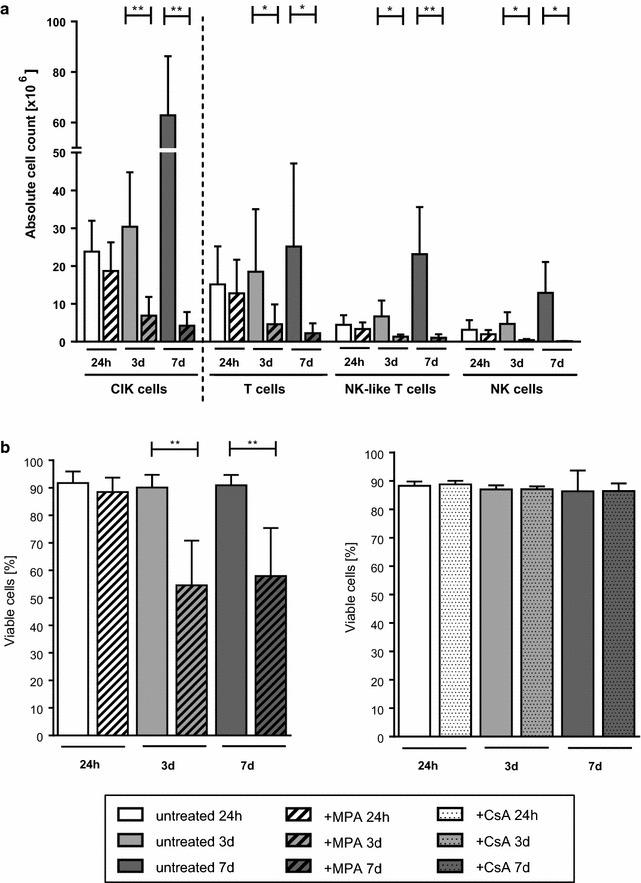
Ex vivo CIK cell expansion upon MPA- and viability with and without MPA/CsA treatment. **a** Proliferation capacity of CIK cells in the presence and absence of MPA for the indicated time-points. Intermediate- (3 days) and long-term (7 days) MPA treatment resulted in an impaired proliferation capacity of CIK cells (p < 0.01), whereas short-term (24 h) MPA exposure had only marginal effect on CIK cell expansion. The most prominent reduction in expansion was seen for NK-like T cells following long-term MPA exposure (p < 0.01). **b** Short-term MPA incubation had no or only marginal effect on CIK cell viability. In contrast, intermediate- and long-term MPA treatment resulted in a significantly reduced CIK cell viability (p < 0.01; n = 6 independent experiments). CsA treatment had no effect on CIK cell viability (n = 4 independent experiments)

CIK cells that were cultivated in the presence of MPA for at least 3 days showed significantly impaired proliferation capacity compared to CIK cells cultivated without MPA over an equal period of time (p < 0.01; Fig. 2a). These differences were also demonstrated for all three CIK cell subgroups following intermediate- and for T- and NK cells following long-term MPA treatment (p < 0.05). Contrary, no effect of CsA treatment on CIK cell proliferation was observed (Additional file 1: Figure S1A). Interestingly, short-term MPA treatment had no or only marginal effect on CIK cell viability. In contrast, intermediate and long-term MPA treatment reduced CIK cell viability by more than 30 % whereas CsA treatment had no significant influence on CIK cell viability (p < 0.01; Fig. 2b).

### Cytotoxic capacity of CIK cells following MPA/CsA treatment

**Fig. 3:**
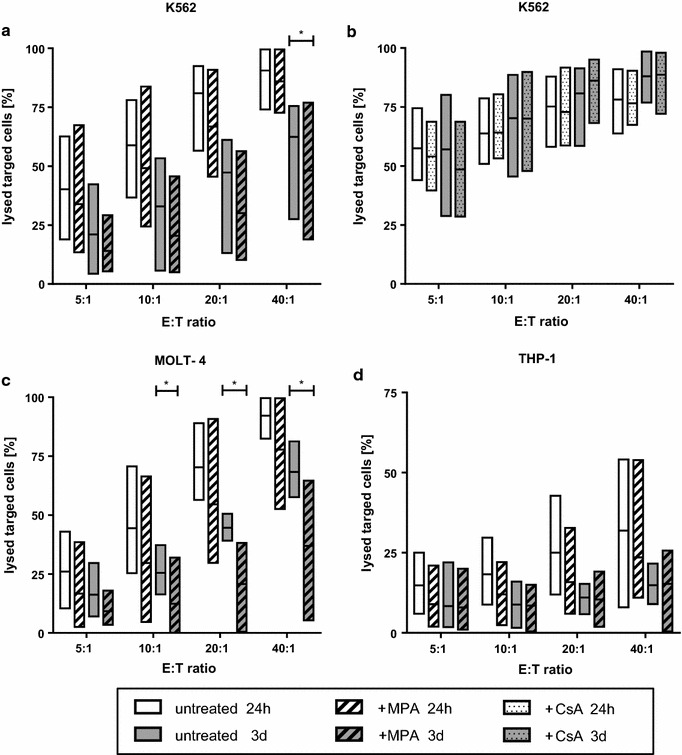
Influence of MPA/CsA exposure on CIK cell cytotoxicity. We investigated a non-radioactive europium release cytotoxicity assay to test the cytotoxicity of MPA treated CIK cells against the ALL cell line MOLT-4 and the AML cell lines K562 and THP-1 and the lytic activity of CsA treated CIK cells against K562. **a** Killing of K562 was influenced only by intermediate-term MPA treatment in the E:T ratio 40:1 with a median killing rate of 62.4 % (SD: 23.3) without and 48.2 % (SD: 24.1) with MPA treatment. **b** CsA treatment of CIK cells did not influence their cytotoxicity against K562 cells. **c** CIK cell mediated MOLT-4 cell lysis was not significantly influenced by short-term (24 h) MPA treatment, whereas intermediate-term (3 days) MPA incubation resulted in reduced killing rates in E:T ratios 10:1, 20:1 and 40:1 (p < 0.05). CIK cell mediated killing was in mean 25.5 % (SD: 9.3), 44.6 % (SD: 5.5), 68.3 % (SD: 10.3) following 3 days without treatment compared to 12.4 % (SD: 13.7), 20.8 % (SD: 17.7), 36.8 % (SD: 27.1) with intermediate-term MPA treatment in E:T ratios 10:1, 20:1 and 40:1, respectively. **d** In contrast to MOLT-4 and K562, the killing efficiency of CIK cells against THP-1 cells was obviously reduced. There was no difference in the lysis of THP-1 target cells of CIK cells with and without MPA incubation. n = 4 independent experiments, respectively

Cytotoxicity of CIK cells with and without MPA treatment was investigated against the ALL cell line MOLT-4 and the AML cell lines K562 and THP-1 whereas CsA treatment was investigated against the cell lines K562 and MOLT-4 (Fig. 3). The cytotoxic effect of MPA treated CIK cells against K562 cells was influenced by MPA exposure, though only in the 40:1 ratio a significant reduction of the killing capacity was obtained **(**p < 0.05, Fig. 3a). In contrast, no differences in the lytic activity of CsA incubated and wildtype CIK cells were observed against K562 (Fig. 3b). Regarding MOLT-4 killing, short-term MPA treatment did not affect target cell lysis compared to untreated control CIK cells (Fig. 3c). However we observed a reduced lysis of MOLT-4 cells by CIK cells treated with MPA for 3 days (intermediate term) in the E:T ratios 10:1, 20:1 and 40:1 (p < 0.05). CsA-treated and wildtype CIK cells showed comparable results in MOLT-4 killing (data not shown). Interestingly, no difference in the lysis of THP-1 target cells of CIK cells with and without MPA incubation were determined (Fig. 3d). Long-term MPA treatment resulted in strong reduction of absolute CIK cell number. Therefore, cytotoxicity testing could not be investigated.

### Changes in receptor expression and cytokine secretion as consequence of MPA treatment

**Fig. 4:**
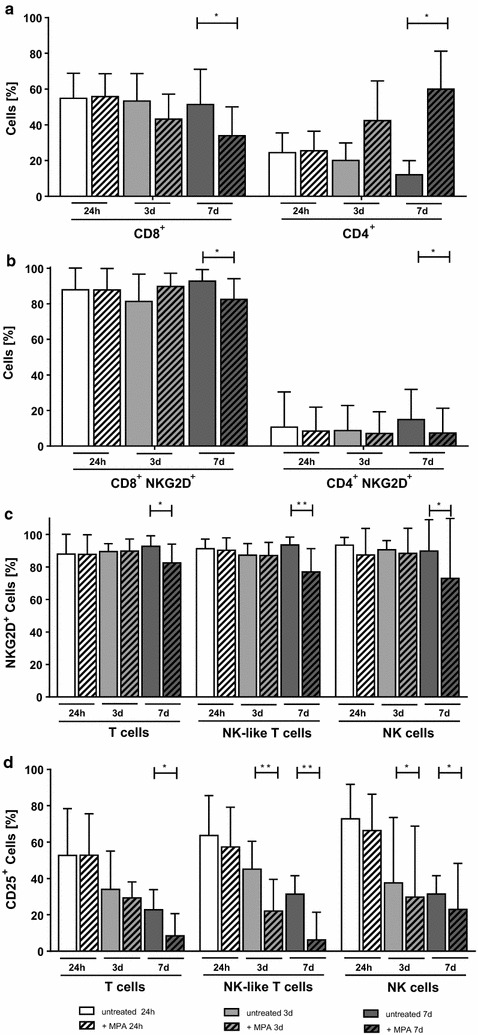
Changes in CIK cell composition induced by MPA treatment. The influence of MPA treatment on receptor expression was analyzed for CD4, CD8, NKG2D and CD25. **a** The percentage of CD8^+^ cells decreased, whereas the proportion of CD4^+^ cells increased. **b**, **c** NKG2D was steadily expressed during short- (24 h) and intermediate-term (3 days) MPA treatment, only showing a reduction after long-term (7 days) MPA exposure. **d** CD25 expression clearly declined on T cells following long-term MPA exposure (p < 0.05) and on NK cells following intermediate- and long-term MPA treatment (p < 0.05). The most significant reduction in CD25 expression was shown for NK-like T cells after intermediate- and long-term MPA exposure. n = 6 independent experiments

Although absolute numbers of T cells declined, we observed an alteration in the CD4/CD8 ratio following MPA treatment of CIK cells. Interestingly, the percentage of CD8^+^ cells decreased, while the percentage of CD4^+^ cells increased (Fig. 4a). Comparing the proportion of CD8^+^ cells following long-term and without MPA treatment revealed a significant decrease in CD8^+^ cells (p < 0.05). In accordance, intermediate and long-term MPA incubation were associated with a statistically significant higher percentage of CD4^+^ cells (p < 0.05 and p < 0.01).

The characterization of NKG2D receptor on CD4^+^ and CD8^+^ cells showed stable expression upon short- and intermediate-term MPA treatment. Only following long-term MPA incubation, downregulation of NKG2D was observed (p < 0.05, Fig. 4b). Further analysis also revealed stable expression of NKG2D on CIK cell subpopulations (T, NK and NK-like T cells) during short- and intermediate-term MPA incubation, whereas reduced NKG2D receptor expression was observed following long-term MPA exposure (T and NK cells: p < 0.05; NK-like T cells: p < 0.01, Fig. 4c).

Regarding CD25, we observed loss of expression when CIK cells were cultivated for longer than the usual culture period of 10 days. However, this reduction in CD25 expression was enhanced following MPA incubation, showing the most pronounced differences for NK-like T cells after intermediate- and long-term MPA treatment (p < 0.01) (Fig. 4d). Interestingly, we also observed a reduction in CD25 expression implicated by CsA treatment. T and NK-like T cells showed a significant reduction of CD25 expression following three (p < 0.01) and 7 days (p < 0.05) of CsA treatment whereas no significant alteration of CD25 expression on NK cells was observed (Additional file 1: Figure S1B). Furthermore, no changes in NKG2D expression in CsA treated CIK cells were examined (data not shown).

**Fig. 5:**
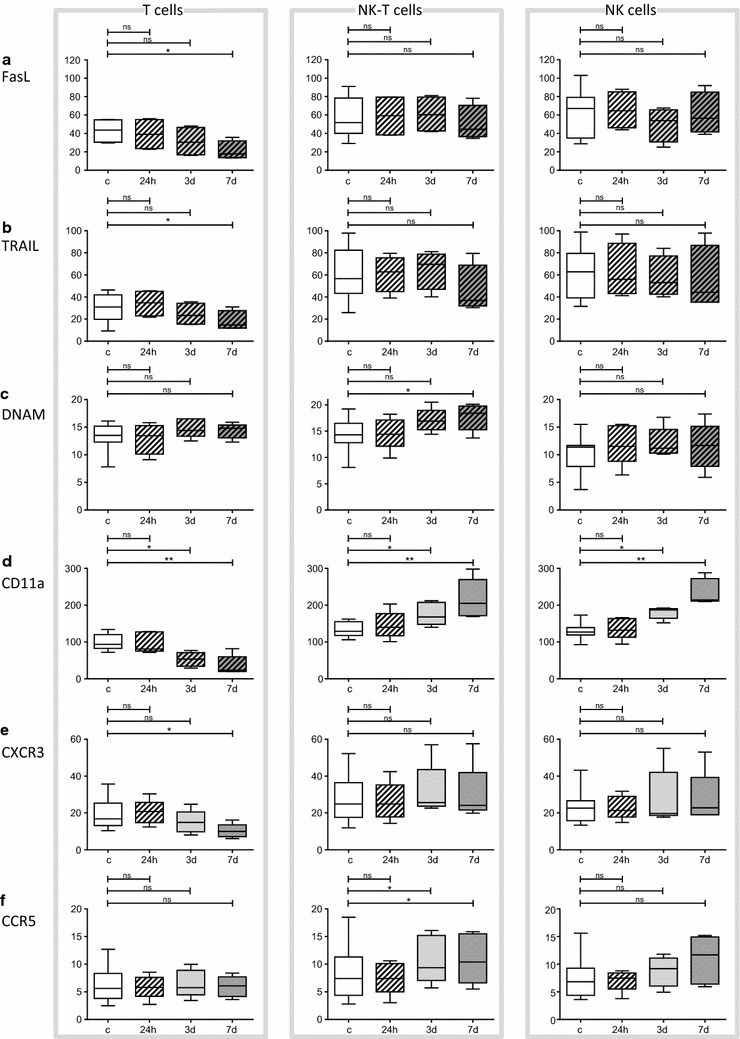
Influence of MPA exposure on receptors involved in CIK cell mediated killing and cell migration. FasL, TRAIL, DNAM, CD11a, CXCR3 and CCR5 were analyzed following short- (24 h), intermediate- (3 days) and long-term (7 days) MPA treatment and all receptors were compared to their individual controls (only 24 h control is displayed). **a**–**c** Analyzing receptors involved in CIK cell mediated killing, we observed a stable expression of FasL, TRAIL and DNAM upon short- and intermediate-term MPA treatment. A reduced expression of FasL and TRAIL on T cells and an increased expression of DNAM following long-term MPA exposure was determined (p < 0.05). **d**–**f** With regard to CIK cell migration, no alteration in the expression of CD11a, CXCR3 and CCR5 upon short-term MPA exposure was observed. CD11a was downregulated on T cells and upregulated on NK-like T and NK cells following intermediate- and long-term MPA treatment (p < 0.05 and p < 0.01). CXCR3 was downregulated on T cells upon long-term MPA incubation (p < 0.05), whereas CCR5 was expressed to a higher extend on NK-like T cells. n = 5 independent experiments

FasL, TRAIL and DNAM, which are mainly involved in target cell killing, were influenced by MPA to a minor extent. Following long-term MPA treatment we determined a decrease in FasL and TRAIL expression on T cells and an increase of DNAM expression on NK-like T cells (p < 0.05, Fig. 5). In contrast, CD11a, CXCR3 and CCR5, which are primarily involved in CIK cell migration, were more susceptible to MPA exposure. Interestingly, the percentage of T cells expressing CD11a was reduced, but increased on NK-like T and NK cells following intermediate- and long-term treatment (p < 0.05 and p < 0.01). Furthermore, T cells showed reduced CXCR3 expression following long-term, whereas NK-like T cells up-regulated CCR5 upon intermediate- and long-term treatment with MPA (p < 0.05).

We also analyzed a panel of cytokines, i.e. IL-8, IFN-γ, MCP-1/CCL2, IL-6, IL-1β and TNFα, following short-, intermediate- and long-term MPA exposure or control incubation. Similar to CD25 expression, cytokine release was attenuated by extended cultivation. In other words, impaired functionality in terms of cytokine release was not primarily influenced by MPA, but rather through the prolonged activation period. The only cytokine significantly reduced after long-term MPA compared to control was TNFα (Additional file 2: Figure S2).

## Discussion

Immunotherapeutic strategies are of increasing interest in the therapy of emerging minimal residual disease or incomplete donor chimerism following HSCT. We and others have previously shown that IL-15 stimulated CIK cells are a promising immunotherapeutic approach for the treatment of patients with impending relapse following allogeneic SCT for acute leukemia or myelodysplastic syndrome [13, 19, 22, 25, 26].

CIK cells consist of a heterogeneous population of CD3^+^ T cells, CD3^+^CD56^+^ NK-like T cells and a minor part of CD3^−^CD56^+^ NK cells [14]. Thereby, CIK cells are able to kill tumor cells via diverse TCR specificities and in a non-MHC-restricted manner [27]. Different studies indicated an anti-tumor effect of CIK cells against various tumor cells mediated by the receptors or ligands NKG2D, TRAIL, DNAM and FasL [16, 28, 29]. In first clinical applications, CIK cell infusions were well tolerated and showed low incidence of GvHD even in the haplo-identical setting, which was postulated to be mainly due to less trafficking of CIK cells to GvHD sites [30], and due to the lack of corresponding ligands or receptors on normal tissues and hematopoietic progenitors.

Beside antileukemic efficacy, CIK cell treatment may be associated with increased risk for developing severe GvHD, especially when being applied in the haploidentical setting and in the early post-transplant period requiring immunosuppressive treatment. Furthermore, even if immunosuppression like MMF is stopped in patients at risk for relapse after HSCT, relevant MPA plasma levels might be present due to intra- and inter-patient variability [31]. Therefore, the influence of immunosuppressive treatment, on the efficacy of cellular interventions needs to be further investigated. Hence, we analyzed the phenotype, survival and cytotoxic capacity of CIK cells during short-, intermediate- and long-term presence of MMF in vitro.

Within this study, we observed that CIK cell cytotoxicity was maintained during short-term MPA exposure, while intermediate- and long-term MPA treatment attenuated proliferation capacity, viability and cytotoxicity. Comparable results were obtained by Brehm et al. [9] for NK cells within the scope of a clinical phase I/II study in short- and long-term MPA treatment. In this study, NK cells were co-incubated with MPA just at the beginning of cytokine activation. In our new approach, we first generated the immunotherapeutic cells and then exposed them to MPA. Eissens et al. [32] and Ohata et al. [33] also described that MPA clearly impeded the outgrowth and the cytotoxic effect of NK cells treated with MPA. Unfortunately, a short-term MPA treatment was not investigated in these two studies. These results are contradicting Shapira et al. [34] who suggested that MMF does not impair GvL-effect or reduce LAK cell activity in mice. However, this might be explained by differing study protocols. Shapira et al. treated mice with MMF for a period of 8 days and afterwards generated autologous LAK cells without further addition of MMF. In contrast, we generated CIK cells according to our study protocol for 10 days and started MPA treatment after harvesting with the intention to simulate in vitro the situation when CIK cells were given to a patient who has therapeutic MPA plasma levels.

Most importantly, none or at most a marginal effect on CIK cell proliferation was seen following short-term MPA incubation. Therefore, our findings are promising, that in case of indispensable immunosuppression post HSCT, the therapy with IL-15 stimulated CIK cells will have at least short-term efficacy. We observed no significant differences of killing relevant receptor expressions of the NKG2D, TRAIL, DNAM and FasL receptors upon short- and intermediate-term MPA exposure. However, we revealed that T cells and NK-like T cells reacted differently upon MPA exposure. CD3^+^CD56^+^ NK-like T cells upregulated receptors relevant for CIK cell killing and migration, whereas CD3^+^ T cells downregulated these receptors. CsA treatment resulted in a non-significant reduction of proliferation and cytotoxicity, only. These results are comparable to Mehta et al. [35] describing that CsA inhibited anti-CD3-mediated degranulation, but did not affect cytotoxicity of CIK cells against tumor targets. Regarding cytokine/chemokine secretion, we observed no significant differences between CIK cells with and without MPA treatment expect for a significant reduction in TNFα secretion. These results are in accordance with Liu et al. [36] who published promising results combining CIK cell therapy with CsA treatment also describing the secretion of various cytokines including IL-2 and IL-8. In contrast, Brehm et al. [9] and Nagy et al. [37] reported a significant reduction in the secretion of IFNγ, IL-6 and other cytokines by NK cells upon MPA treatment, but here immune cells were co-incubated with MPA already during cultivation time.

Following infusion, CIK cells are described to accumulate and persist in tumor sites, resulting in tumor eradication [30]. Wang et al. [38] analyzed CIK cell homing of ^18^F-FDG labeled CIK cells in leukemia patients by PET/CT tracking. They figured out, that 1 h following CIK cell infusion, the majority of CIK cells accumulated in the lungs, followed by a migration into brain, heart, liver and spleen at the time points 4 and 8 h post CIK cell therapy. Furthermore, tendencies of CIK cell homing into the bone marrow were shown. In the study described herein, we observed that short-term MPA incubation had no pronounced effect on proliferation, viability, cytotoxicity and CIK cell composition. Linking this information to the homing parameters analyzed by Wang et al., we might speculate that during a window of 24 h where hardly any influence of MPA on the CIK cells was seen, CIK cells might migrate to tumor sites and achieve a cytotoxic effect.

## Conclusions

In conclusion, already generated IL-15 CIK cells that were co-incubated with MPA for at least 3 days showed significantly impaired proliferation capacity, restricted viability, alterations in receptor expression and a reduction in their cytotoxic capability compared to CIK cells that were cultivated without MPA. Interestingly, CsA treatment had no significant influence on CIK cell viability and the cytotoxic potential against K562. However, a short-term MPA incubation had only marginal or reduced effect on CIK cells. The favored strategy is to avoid immunosuppression in patients who received CIK cell immuno-therapy. However, our findings showed, that even in patients with immunosuppression e.g. for treatment of GvHD, CIK cell treatment may have at least short-term efficacy.

## Additional files

10.1186/s12967-016-1024-4 Ex vivo CIK cell expansion and CD25 expression upon CsA treatment. (A) CsA treated CIK cells showed comparable expansion rates compared to wiltype CIK cells. (B) CD25 expression significantly decreased on T and NK-like T cells following 3 and 7 days of CsA incubation (day 3: p < 0.01 and day 7: p < 0.05).
10.1186/s12967-016-1024-4 Impact of MPA treatment on cytokine secretion. Analyzing the secretion of IL-8, IFNγ, MCP-1/CCL2, IL-6, IL-1β and TNFα, we only determined a significant decrease in TNFα secretion upon long-term MPA exposure (p < 0.01). n = 4 independent results in triplicates. Abbreviations: c = control, +MPA = following MPA treatment.

## Abbreviations

APC: allophycocyanin
APC-A700: APC-Alexa Fluor 700
APC-A750: APC-Alexa Fluor 750
CIK: cytokine-induced killer
CsA: Ciclosporin A
FITC: fluorescein–isothiocyanate
ECD: phycoerythrin-Texas Red^®^
GvL/T: graft-versus-leukemia/tumor
DLI: donor lymphocyte infusion
GvHD: graft-versus-host disease
GMP: good manufacturing practice
HSCT: hematopoietic stem cell transplantation
KO: krome orange
MMF: mycophenolate mofetil
MPA: mycophenolic acid
MRD: minimal residual disease
NK: natural killer
PBMC: peripheral blood mononuclear cells
PB: pacific blue™
PC-5: phycoerythrin–cyanine-5
PC-7: phycoerythrin–cyanine-7
PE: phycoerythrin
