# Supplementary figures and images for: In-vitro influence of mycophenolate mofetil (MMF) and Ciclosporin A (CsA) on cytokine induced killer (CIK) cell immunotherapy
Source: J Transl Med. 2016 Sep 13;14(1):264. doi: 10.1186/s12967-016-1024-4 (PMC5020454; doi:10.1186/s12967-016-1024-4)

## Slide 1
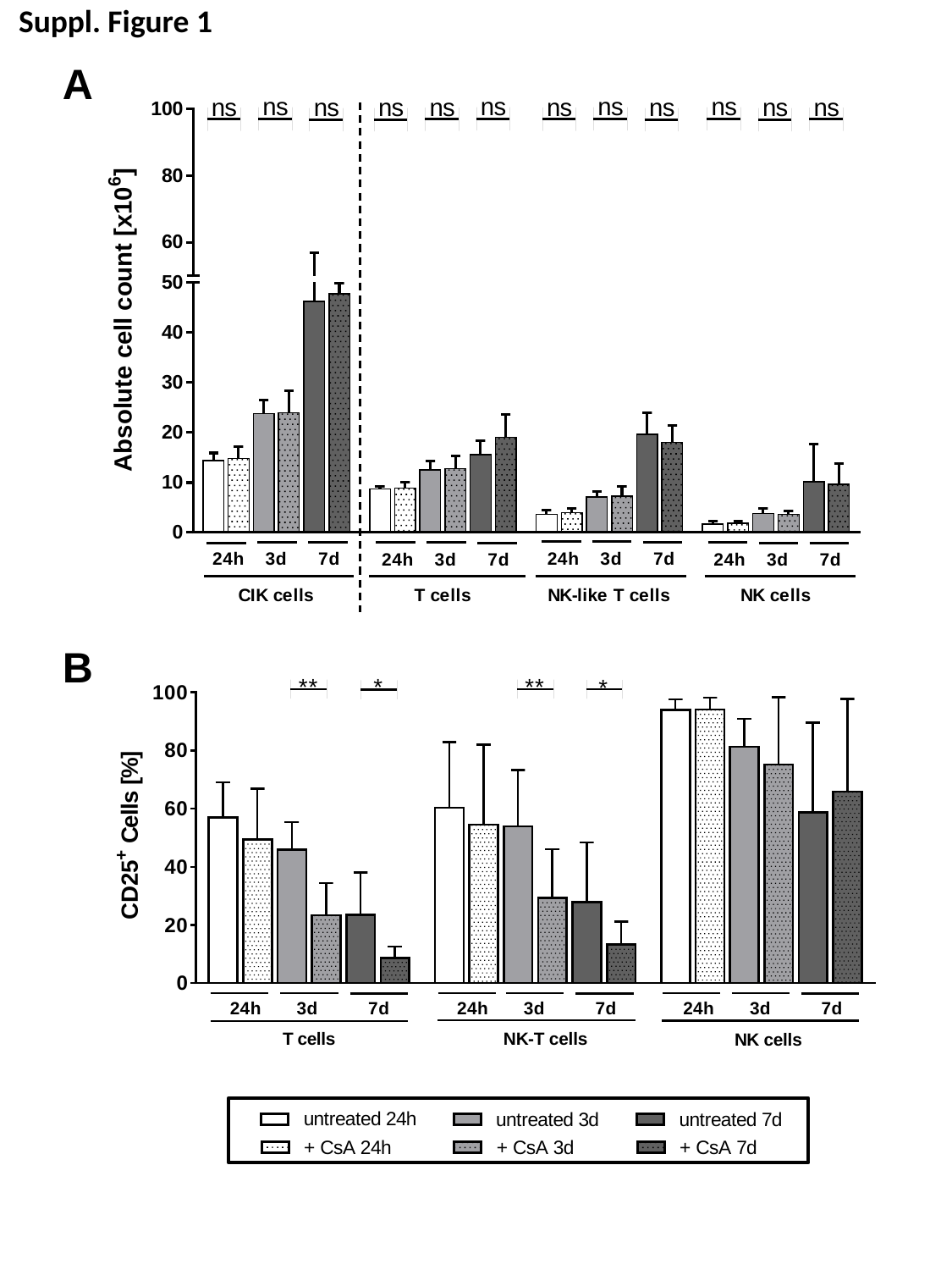

Suppl. Figure 1
A
B

Supplement: Supplementary file 1 — 10.1186/s12967-016-1024-4 Ex vivo CIK cell expansion and CD25 expression upon CsA treatment. (A) CsA treated CIK cells showed comparable expansion rates compared to wiltype CIK cells. (B) CD25 expression significantly decreased on T and NK-like T cells following 3 and 7 days of CsA incubation (day 3: p < 0.01 and day 7: p < 0.05). [file 12967_2016_1024_MOESM1_ESM.pptx]

## Slide 1
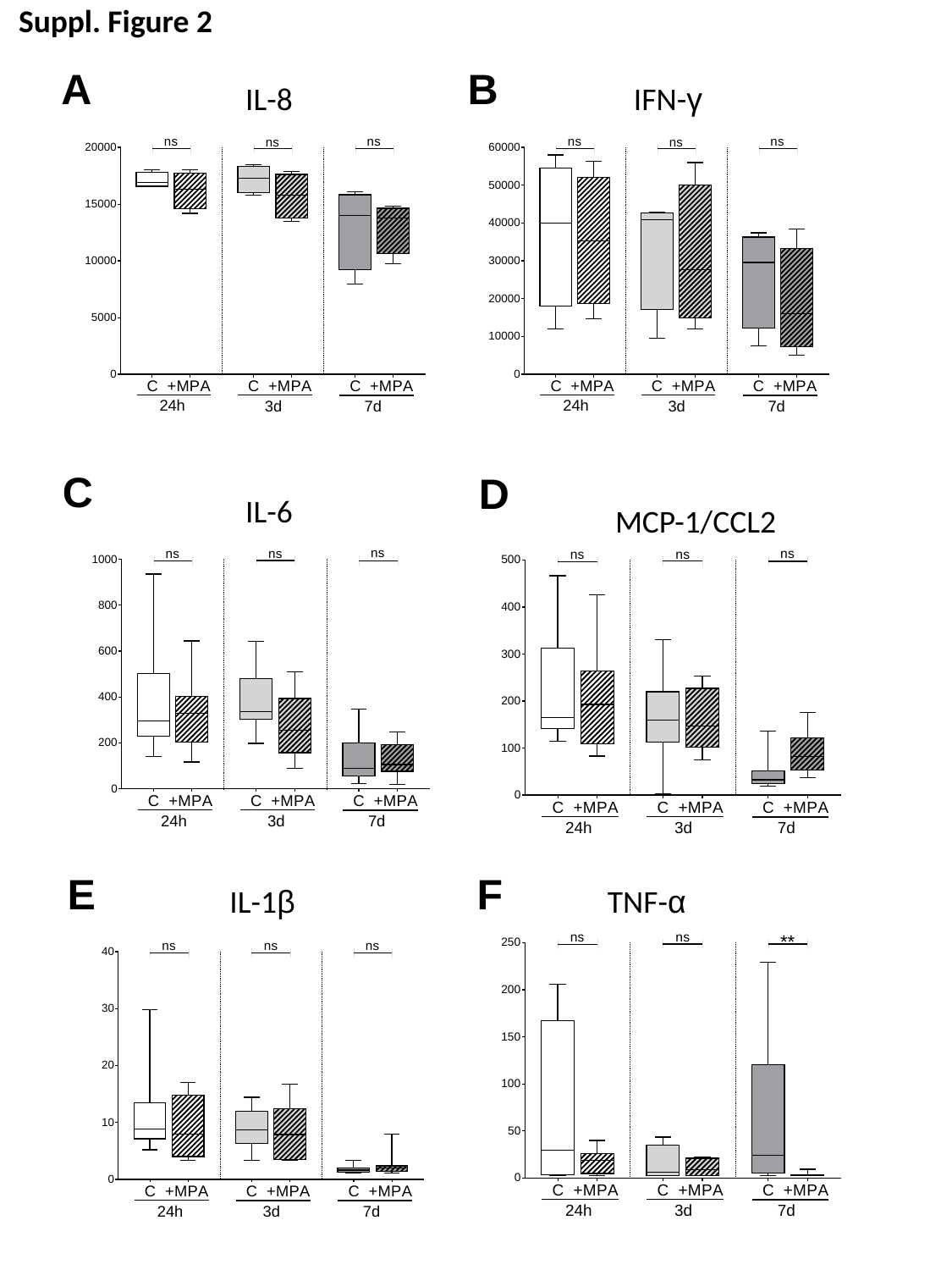

Suppl. Figure 2
A
B
IL-8
IFN-γ
C
D
IL-6
MCP-1/CCL2
E
F
IL-1β
TNF-α

Supplement: Supplementary file 2 — 10.1186/s12967-016-1024-4 Impact of MPA treatment on cytokine secretion. Analyzing the secretion of IL-8, IFNγ, MCP-1/CCL2, IL-6, IL-1β and TNFα, we only determined a significant decrease in TNFα secretion upon long-term MPA exposure (p < 0.01). n = 4 independent results in triplicates. Abbreviations: c = control, +MPA = following MPA treatment. [file 12967_2016_1024_MOESM2_ESM.pptx]
